# Improvements in Capacity Among Community Coalitions Receiving Technical Assistance Through the Coalition Check-Up

**DOI:** 10.1007/s11121-026-01941-z

**Published:** 2026-06-24

**Authors:** Yvonne Gaddy, Sarah M. Chilenski, Rebecca Wells, Louis D. Brown

**Affiliations:** 1 Department of Health Promotion and Behavioral Sciences, The University of Texas Health Science Center at Houston, School of Public Health, El Paso, TX USA; 2 Edna Bennett Pierce Prevention Research Center, Pennsylvania State University, University Park, PA USA; 3 Department of Management, Policy and Community Health, The University of Texas Health Science Center at Houston, School of Public Health, Houston, TX USA

**Keywords:** Community coalitions, Low intensity technical assistance, TA, Coalition capacity, Coalition Check-up

## Abstract

Community coalitions are central to the prevention delivery system, yet their ability to implement and sustain effective strategies often depends on technical assistance (TA). TA typically strengthens coalition capacity, but many coalitions lack connection to TA, and it is costly to sustain. Low intensity TA models that support coalition capacity exist but have not been rigorously evaluated. This study examines the Coalition Check-Up (CoCU) as a low intensity TA model to strengthen coalition capacity. Using up to 3 years of data, we tested whether capacities selected by coalitions during each CoCU implementation year improved over time. Thirty-two coalitions in Pennsylvania and Missouri received TA to review their summary report on data collected yearly over 3 years and a follow-up after year 3. Each year, coalitions used their report to prioritize a capacity for action planning following the CoCU TA model. Analyses examined data from multiple baselines (i.e., before action planning at year 1, year 2, and year 3) and subsequent data collection timepoints to test for longitudinal change on prioritized capacities. Coalitions receiving TA through the CoCU model demonstrated measurable improvements over time. Capacities prioritized in year 1 significantly improved across the following years measured (*β* = 0.13, 95% confidence interval (CI) [0.004, 0.26]; *p* = .04), as did capacities selected in the second year (*β* = 0.20, CI [0.02, 0.37]; *p* = .03) and the third year (*β* = 0.61, CI [0.16, 1.07]; *p* = .01). Findings suggest that a low intensity TA model like CoCU has the potential to guide coalitions toward strategic capacity development, ultimately enhancing their ability to deliver impactful prevention initiatives.

**Supplementary Information:**

The online version contains supplementary material available at 10.1007/s11121-026-01941-z.

Community coalitions are a vital part of the prevention delivery system, particularly in supporting community-led efforts for systemically marginalized populations (Flewelling & Hanley, 2016; Wandersman et al., 2008). By bringing together individuals and organizations from diverse community sectors to work together toward a common goal, coalitions pool complementary skills, expertise, and resources essential for effective prevention activities (Butterfoss & Kegler, 2002). Through their established presence, coalitions are well positioned to identify local needs and develop tailored efforts to improve community health and wellness (Nagorcka-Smith et al., 2022). However, evidence of coalition effectiveness emerged primarily after comprehensive technical assistance (TA) and evidence-based practices (EBPs) were incorporated into coalition models (Hallfors et al., 2002; Hawkins et al., 2014; Spoth et al., 2004, 2013). TA is critical for EBP implementation because EBPs typically require greater technical expertise, coordination between partners, and monitoring than do home-grown practices (Cameron et al., 2001; Rohrbach et al., 2006)*.* Past research found that coalitions using TA were more likely to utilize EBPs (Fagan et al., 2012), as well as implement them with higher fidelity (Chinman et al., 2014) and achieve better community health and behavioral-related outcomes (Hawkins et al., 2014; Rowhani-Rahbar et al., 2023). Nonetheless, evidence indicates that TA may not translate into effective dissemination, implementation, and sustainment of prevention efforts when underlying coalition capacity is limited (Scott et al., 2022).

Researchers supporting substance use prevention coalitions have identified TA as a key factor in helping coalitions manage the complexities of coalition operations to support community change activities. Ideally, TA typically involves individualized, collaborative, hands-on interactions in which TA providers support coalitions in building capacity for long-term effectiveness and sustainability (Chilenski et al., 2016; Florin et al., 1993; Katz & Wandersman, 2016). Providers use approaches such as data-driven decision-making support, evaluation and performance feedback, and guidance for problem-solving and action planning to meet coalition priorities (Chilenski et al., 2019; Metz et al., 2020; Scott et al., 2022). Research indicates that using a collaborative approach to implement these techniques may bolster coalition capacity by empowering coalitions rather than directing decisions (Chilenski et al., 2016; Metz et al., 2020). Prior evidence from a range of health-related contexts also suggests that TA that actively involves organizational members in all stages of capacity building can enhance their shared vision and mutual accountability for ongoing improvements and sustainable changes (Metz et al., 2020).

Past research demonstrates that intensive TA can substantially strengthen community coalitions by improving organizational functioning—such as developing bylaws and mission statements, recruiting members, and running efficient meetings (Florin et al., 1993)—as well as sustaining evidence‑based programs and enhancing fidelity of program implementation (Watson-Thompson et al., 2013). Studies of models such as PROSPER further highlight that individualized, hands‑on TA fosters trust, collaboration, commitment, and role clarity, enabling coalitions to adapt to challenges and pursue long‑term community change (Chilenski et al., 2021). Yet even if intensive TA helps accelerate short-term outcomes, coalitions ultimately need to develop internal capacity to sustain progress once external support diminishes (Chilenski et al., 2021).

Intensive TA typically involves continuous engagement over extended periods, frequent contact (e.g., ongoing coaching, tailored consultation, and site visits), and support from multiple specialized staff, often at approximately 25% FTE per coalition (Chilenski et al., 2021). For many coalitions, however, intensive TA may not be a viable option, as it requires substantial investments in time, funding, and skilled personnel—resources that are often scarce, especially in under-resourced communities (Chilenski et al., 2021; Dunst et al., 2019; Feinberg et al., 2008c; Michaud et al., 2022). Funding models that prioritize start-ups further exacerbate inequities. New coalitions may receive more TA, while coalitions that have existed for more years face declining access despite ongoing needs (Feinberg et al., 2008a; Foster-Fishman et al., 2001; Raynor, 2021). Although continuous, tailored TA across coalition life stages likely predicts stronger functioning and long-term sustainability (Chilenski et al., 2021; Scott et al., 2022), evidence on TA effectiveness remains mixed and varies by type and intensity of support, with limited attention to dosage (Acosta et al., 2013; Chilenski et al., 2021; Feinberg et al., 2008b). As a result, it remains unclear whether greater TA intensity yields proportionately greater benefits, or whether low intensity approaches may achieve comparable gains on coalition outcomes. These realities underscore that relying solely on intensive TA is neither scalable nor equitable. Low intensity and hybrid TA models are therefore essential. By delivering meaningful support at a fraction of the cost, such TA models expand access, reduce disparities, and provide a viable pathway for strengthening coalition capacity in the long term (Katz & Wandersman, 2016; Scott et al., 2022). Understanding how low‑intensity approaches can effectively strengthen coalitions requires a conceptual framework that clarifies the role TA plays within the broader implementation systems.

The Interactive Systems Framework for Dissemination and Implementation (ISF) provides such a foundation, illustrating how TA functions as a critical support mechanism in public health and community coalition work. The ISF outlines three systems that must function synergistically to address the gap between science and practice in community public health strategies (Wandersman et al., 2008). The synthesis and translation system converts research into user-friendly formats for practitioners, whereas the delivery system implements evidence-based practices in real-world settings (Katz & Wandersman, 2016). Connecting these two systems is the support system, where TA operates to help the delivery system incorporate research into practice (Katz & Wandersman, 2016). The Coalition Check-Up model is situated within this prevention support system and provides structured, collaborative TA to help coalitions take action on prioritized capacity characteristics. In doing so, this model was theorized to strengthen the conditions necessary to effectively implement prevention initiatives (Wandersman et al., 2008).

As a support-system intervention, the Coalition Check-Up reflects broader theory and evidence regarding the role of TA in building coalition capacity. Within the prevention support system, TA can serve as a capacity-building strategy for substance use prevention coalitions (Gayles et al., 2024). With the help of a TA provider, coalitions can prioritize and address areas for improvement based on readiness and alignment with coalition goals, thereby building capacity to impact youth and community outcomes (Wandersman & Florin, 2003; Watson-Thompson et al., 2013). Although the importance of TA is well established, practice settings are typically under-resourced to implement existing evidence-based TA models, which are resource intensive. While low intensity technical assistance models to support coalition capacity exist, few have been rigorously evaluated (Brown et al., 2021). This article examines the Coalition Check-Up as a low intensity TA model to strengthen coalition capacities.

## The Coalition Check-Up TA Process

The Coalition Check-Up (CoCU) (Brown et al., 2021) was designed as a low intensity, data-driven TA system that is broadly applicable across community substance use prevention coalition models. The CoCU supports coalition strategic planning by addressing capacity gaps prioritized by coalitions as areas in need of improvement. A CoCU TA provider works with the coalition to (a) conduct validated assessments of coalition capacity, including for program implementation (Brown et al., 2012; Feinberg et al., 2008a, b, c); (b) review the assessment feedback report; (c) identify priority areas for improvement; (d) integrate evidence-informed practices into an action plan that addresses prioritized issues; and (e) monitor and support action plan implementation. To quickly build strong collaborative relationships, ground planning in each coalition’s goals, and enhance motivation and confidence in success, TA providers employ motivational interviewing techniques in all coalition interactions (Chilenski et al., 2026; Romano & Peters, 2016).

## The Current Study

Building on evidence that TA can bolster coalition capacity under certain conditions (Chilenski et al., 2016; Katz & Wandersman, 2016), we examined whether a low intensity TA model supported enhancements in specific capacities coalitions selected for action planning. More precisely, we hypothesized that the capacities prioritized each year during the CoCU intervention would improve in later assessments of coalition capacity. To test this, we analyzed changes in the capacities selected each implementation year using up to 3 years of coalition and program assessment data from the CoCU study (Brown et al., 2021).

## Methods

### Study Participants

Thirty-two community coalitions in Pennsylvania (84%) and Missouri (16%) participated in the CoCU TA and training process as part of a larger study of coalitions (Brown et al., 2021) recruited by Evidence-based Prevention and Intervention Support (EPIS) TA providers in the Edna Bennett Pierce Prevention Research Center at Penn State University. Coalitions were eligible if they had a designated coordinator, had been in operation for at least 1 year, held meetings at least quarterly with multiple community sectors in attendance, and supported drug prevention efforts or had secured funding to do so. Coalitions had to agree to the annual assessment process and randomization to either the CoCU TA and training process or a control condition. The current study only includes coalitions assigned to receive the CoCU intervention. At study initiation, these coalitions—ranging in age from 1 to 37 years (mean = 11.3 years; *SD* = 8.6)—operated mostly in rural or mostly rural locations (59%) and 59% were receiving funding. The average coalition size ranged from 36 members (year 1) to 57 members (year 3). The average population in coalition service areas was 81,577.1 (*SD* = 98,787.9), mostly White (84%) and Black (9.4%). An average of 12% of the local population had incomes below the federal poverty threshold compared to the national average of 11.4% (United States Census Bureau, 2022). The most commonly employed coalition model was Communities That Care (CTC) (25%); other models included Drug-Free Communities (DFC) (22%), Strategic Prevention Framework (6%), PROmoting School-community-university Partnerships to Enhance Resilience (PROSPER) (3%), other (9%), multiple models combined (19%), or no model (16%). Study procedures were approved by Penn State University’s Institutional Review Board as the single IRB of record.

### Data Collection

Starting with the 2020–2021 school year, data collection occurred four times: at the start of each of the three intervention years and a follow-up 1 year later, at the end of year 3. Year 1 data collection was completed before randomization to the intervention occurred. The current analyses were derived from the 45–60-min interviews with coalition leaders, the 30–45-min interviews or surveys among program coordinators, and the 20–30-min self-administered surveys completed by coalition members during each data collection. Questions covered specific characteristics of their coalition, such as coalition resources, processes, structure, and program implementation. Both surveys and interviews collected quantitative responses; questions in each were directed to respondents according to their role and coalition-related activities (i.e., member, coalition leader, program coordinator). In our experience, people often prefer interviews over a long online survey. Thus, we offered interviews for the coalition leaders and program coordinators. Starting at wave 2, we shortened the program coordinator data collection and began offering respondents the option of completing it via survey or interview. See supplemental materials for additional information on survey and interview sample items. Participation was voluntary, with incentives ranging from $10 to $40. Across the four data collection points, member survey participation rates ranged from 55 to 63%; program coordinator participation ranged from 91 to 99%; and 97% to 100% among coalition leaders. Consistent with prior research (Butterfoss & Kegler, 2002), we define members as individuals who participate in coalition activities and decision making and who may represent themselves or an organization or sector. A coalition leader is typically the individual who facilitates coalition activities. This may be one person or a team of people. We refer to this role as coalition leader throughout. Program coordinators typically manage day-to-day implementation of one or more programs connected to the coalition. Individuals can serve in one or more roles and were asked to participate in the data collection that matched their role or roles.

Table 1 shows the number of coalitions participating at the beginning of each implementation year. At the start of years 1 and 2, all 32 coalitions were active in the intervention. At year 3, 29 coalitions were active, as one coalition dropped from the study during year 2 for unknown reasons, and three coalitions merged into one.

**Table 1:** Number of coalitions actively participating during each CoCU implementation year

Number of coalitions	CoCU implementation year		
	Year 1 model	Year 2 model	Year 3 model
Active *n*^a^	32	32	29
Reviewed their feedback report	32	29^b^	26^c^
Identified a characteristic of capacity for action planning (*n*)	32	26^b^	25^c^

^a^Number of coalitions participating in the intervention at the start of each year

^b^Year 2: 6 coalitions did not identify a characteristic (3 paused activities due to leadership changes or reorganization; 3 merged into one before identifying a characteristic)

^c^Year 3: 4 coalitions did not identify a characteristic (3 disbanded shortly after reviewing their report; 1 disbanded before identifying a characteristic)

### Coalition Check-Up Intervention Procedures

After completing each data collection, we aggregated survey and interview responses to the coalition-level and provided coalitions with a feedback report synthesizing data across 68 coalition capacity and program characteristics (see supplemental materials for details) (Chilenski & Brown, 2021). Data from each sample was then used to create constructs measured by a single reporter (i.e., member, coalition leader, program coordinator). Of the 68 characteristics, the report covered 49 characteristics of coalition capacity identified in prior research as important for coalition functioning and effectiveness (Brown et al., 2012; Chilenski et al., 2025; Chinman et al., 2005; Feinberg et al., 2008b). For example, *member recruitment* (1 item) asked about the difficulty in getting the “right” people with skills, talents, and needed connections to join the coalition. *Developed mission and communications plan* (1 item) asked to what degree the coalition had finalized a vision and mission statement, and tools and strategies to communicate with outside groups*.* The remaining 19 of the 68 characteristics focused on programs connected to the coalition (Brown et al., 2010; Durlak & DuPre, 2008; Foster-Fishman et al., 2001; Wandersman et al., 2008). *Coalition connection* (3 items), for example, asked how often program staff attend coalition meetings, report progress to coalition members, and seek advice from the coalition. Items were summarized using tables, charts, and graphs with a color-coded benchmarking system grounded in prior coalition research and practice-informed best practices (Brown et al., 2012; Chilenski et al., 2025; Chinman et al., 2005; Feinberg et al., 2008b) and on the mean score across coalitions in the study (displayed on graphs) to indicate areas of strength, neutrality, or growth. Coalitions could easily visualize their strengths and potential areas for improvement, examine change from the previous year, and situate their performance relative to peer coalitions.

The CoCU intervention started with a meeting between the assigned TA provider and the coalition leader, during which the TA provider emphasized the leader’s role as an expert in their coalition and community. The TA provider then introduced the feedback report, orienting the coalition leader to its structure and highlighting the color-coded dashboards aligned with each component of the CoCU logic model. The coalition leader was invited to identify where to begin the data review. At a minimum, TA providers and coalition leaders reviewed the dashboard of each logic model component together. The discussion focused on guiding coalition leadership through the feedback report to identify strengths and potential characteristics for growth (Chilenski et al., 2026). TA providers took notes and, by the end of the review, leaders had generated a list of prioritized characteristics. In most cases, the process naturally yielded 5–7 characteristics; when it did not, TA providers facilitated further discussion to refine the list to this target range. Leaders then discussed the 5–7 prioritized characteristics with the full coalition, which collectively selected one characteristic for action planning. Priority selection was guided by coalition leaders’ and members’ interpretation of the feedback data, with characteristics chosen based on perceived importance, feasibility, and relevance to the coalition’s goals rather than externally defined criteria. The TA provider facilitated the process but ensured all decisions were coalition-driven and encouraged leaders and members as experts to interpret the report’s findings.

Approximately 1 month after the coalition prioritized a characteristic for the implementation year, the TA provider met with the coalition to begin the evidence-informed action-planning process. The process started by reviewing the prioritized characteristic and the challenges it presented and by setting improvement goals. Coalitions then used the Coalition Check-Up Guidebook (Brown & Chilenski, 2022), which defined each characteristic included in the feedback report and provided a list of research- and practice-informed strategies that are likely to strengthen those areas. Guided by these materials, coalitions discussed strategies linked to their prioritized characteristic and brainstormed to identify the approach that best aligned with their goals. For example, strategies to strengthen community knowledge of the coalition’s work include creating a regular newsletter and sending it to stakeholders and providing locally relevant data through brief, easy-to-understand formats such as infographics. Strategies to promote member recruitment include delegating outreach efforts to those who are most familiar or most likely to be influential among potential members and ensuring coalition meetings are scheduled at convenient and accessible times and locations. Together, members reflected on the pros, cons, and resource demands for each potential strategy. The group then determined the best action plan strategy, developed a step-by-step plan to implement the strategy, assigned responsibilities, and defined success metrics (Chilenski et al., 2024).

Once the plan was completed and adopted, the TA provider monitored and supported implementation using an action plan progress tracker (Fausey et al., 2024), meeting with coalitions twice over the next 6 months to discuss progress and help coalitions address any challenges in implementing the action plan. The TA provider also met with coalition leaders virtually or by phone approximately monthly to review progress, discuss concerns, and plan next steps. After the first year, the process began again with the next data collection and feedback report. Throughout the process, the TA provider used motivational interviewing techniques to cultivate collaboration and decision-making. CoCU fidelity monitoring was continuous over the 3 years.

As designed, the CoCU steps described above were completed through 16 TA provider–coalition/coalition leader contacts over the course of 1 year (Chilenski et al., 2026). Those included one monthly phone or videoconference contact with coalition leaders and four annual in-person or videoconference contacts with the full coalition, depending on the coalition’s usual meeting format. For coalitions that used a hybrid format, TA providers attended the meeting in person. Other additional, as-needed communications in the form of emails or phone calls also occurred, typically between the TA provider and the coalition leader.

Table 1 illustrates the number of coalitions that reviewed their feedback report and identified a characteristic for action planning. At year 1, all 32 coalitions reviewed their report and identified a characteristic. At year 2, 29 of 32 coalitions reviewed their report; 26 identified a characteristic. Three coalitions paused study participation due to leadership changes or reorganization during the feedback report review process; three others reviewed their report but merged into one before prioritizing a characteristic—the merged coalition did not participate until year 3. At year 3, 26 of 29 coalitions reviewed their report; 25 selected a characteristic. Three coalitions disbanded shortly after reviewing their report; one disbanded before identifying a characteristic. We report here on the coalitions that identified a characteristic for action planning during the respective implementation year: year 1 (*n* = 32), year 2 (*n* = 26), and year 3 (*n* = 25).

### Measures

Outcomes measured in this study corresponded to the characteristics coalitions prioritized for action planning each year. For the current study, we grouped the characteristics into five general categories of coalition capacity. Similar to previous research (Nargiso et al., 2013), we define these categories as follows: (1) mobilization—capacity to harness the collective power of a wide range of community partners to work together toward common goals; (2) structure—capacity to establish a formalized structure to maximize operations; (3) leadership—capacity for leadership that promotes vital action for coalition operations; (4) teamwork—capacity to foster synergistic collaboration; and (5) planning and implementation—capacity to establish plans to support optimal implementation and sustainability of prevention efforts. Details on all characteristics are provided in the supplemental materials, including definitions, sample items, and response options.

We identified various measures as potential covariates—coalition age, state (Pennsylvania or Missouri), coalition model employed (e.g., Communities That Care), whether coalitions received funding for prevention efforts, and coalition size. All measures corresponded to the time coalitions were recruited to the study, except coalition size, which was based on leaders’ estimates of the number of members who attended at least two coalition meetings during the 12 months prior to each data collection. Specific demographics in coalitions’ service areas were also identified as possible covariates. Community poverty rates, population, urban or rural status, and race were determined using 2020 Census data (2020), the Center for Rural Pennsylvania (2021), or the National Center for Education Statistics (2020). Weighted averages were calculated for coalitions whose service area extended across more than one region.

### Data Analysis

Data analyses at the coalition level—given our focus on coalition-level outcomes—were conducted using proc mixed in SAS™ Enterprise Edition 3.81 (SAS Institute Inc., 2022). The effect of time on selected priorities was our independent variable, and the scores for each coalition’s priority in a given year were the dependent variables. First, we created coalition-level scores for all coalition capacity measures by averaging individual participant responses together at the coalition level at each data collection. Given the variation in scaling for each capacity measure, coalition capacity scores for all measures were standardized to a grand mean of 0 and standard deviation of 1 across coalitions and the four data collection timepoints, and all scales were scored such that higher scores represented more favorable outcomes.

We then created separate models for each CoCU implementation year—1, 2, and 3—to model the effect of time on scores for the selected characteristics. Data collected at the start of each year served as the respective model’s baseline to test for longitudinal changes in scores across subsequent years. Specifically, year 1 data functioned as the baseline for the year 1 model to test changes in characteristics selected during implementation year 1. Year 2 scores were the baseline for implementation year 2; year 3 scores were the baseline for implementation year 3. Model fit indices, including the AIC and BIC, were compared across different parameterizations of time, including the empty model (i.e., model specifying only a random intercept, no change over time), a linear model, and a polynomial growth model to determine the best longitudinal model for each year. We found that the random intercept model with time as a linear predictor was best to model the effect of time on scores for each implementation year, as shown in Table 2.

**Table 2:** Model fit statistics for the best growth model to test changes over time on the characteristics coalitions selected for action planning

	Random intercept only	Random intercept, time linear^a^	Random intercept, time linear with random slope/time
Year 1 model fit statistics			
− 2 Log likelihood	297.9	293.8	292.6
AIC	303.9	301.8	304.6
BIC	308.3	307.6	313.4
Degrees of freedom	3	4	6
Parameter estimates			
Fixed effects			
Time: linear slope term		.13^*^	.13
Random variance: intercept	−.32	−.49	−.49
Year 2 model fit statistics			
− 2 Log likelihood	178.6	173.6	173.6
AIC	184.6	181.6	183.6
BIC	188.3	186.7	189.9
Degrees of freedom	3	4	6
Parameter estimates			
Fixed effects			
Time: linear slope term		.20^*^	.20^*^
Random variance: intercept	−.26	−.64	−.64
Year 3 model fit statistics			
− 2 Log likelihood	137.9	131.2	
AIC	143.9	139.2	
BIC	147.6	144.1	
Degrees of freedom	3	4	
Parameter estimates			
Fixed effects			
Time: linear slope term		.61^*^	.61^*^
Random variance: intercept	−.51	− 2.04	− 2.04

^a^Best fitting model, years 1, 2, and 3

*p <.05

We then tested the identified covariates as potential confounders in each model. Each covariate was entered into the respective model one by one to measure its impact on the effect of time and was kept in the model if it changed the effect of time by more than 10% regardless of significance (VanderWeele, 2019). However, none of the covariates met this requirement in any of the separate models; hence, no covariates were kept in the models.

## Results

Table 3 provides the frequency with which each characteristic was selected for action planning across the three years. Selections varied by year and could be repeated; the most commonly selected capacities were member recruitment (9 times total), community’s knowledge of the coalition’s work (8 times), inclusion of community residents (6 times), and school support for the coalition (6 times), all of which were characterized as mobilization. Clarity of internal coalition roles and community support for the coalition, also in the mobilization category, and developed mission and communications plan, in the structure category, were each selected five times total. Table 4 lists the characteristics coalitions never selected during the study. Table 3 Supplemental Information and Table 4 Supplemental Information provide additional details on the characteristics coalitions selected during the study and those never selected, respectively.

**Table 3:** Frequency with which characteristics of coalition capacity were selected for action planning

Category (selected/total) Characteristic	Frequency selected		
	Year 1 ***n*** = 32	Year 2 ***n*** = 26	Year 3 ***n*** = 25
1. Mobilization (8/9)			
Member recruitment	2	4	3
Community knowledge of the coalition's work	2	3	3
Inclusion of community residents	4	2	0
School support for the coalition	2	2	2
Clarity of internal coalition roles	3	1	1
Community support for the coalition	2	1	2
Number of member roles	2	1	1
Member involvement	0	0	2
2. Structure (7/21)			
Developed mission and communications plan	3	2	0
Members oriented to coalition’s implementation model	2	0	1
Number of committee/subcommittee/workgroup meetings	0	1	1
Formalized structure for consistent implementation of policies and procedures	1	0	1
Attendance	1	0	0
Member collaboration with different organizations	1	0	0
External partner role clarity	1	0	0
3. Leadership (2/2)			
Number of formal leadership positions	0	1	0
Leadership transitions	0	1	0
4. Teamwork (3/5)			
Cohesion	1	2	1
Meeting focus	2	0	0
Efficient meetings	0	1	0
5. Planning and implementation (6/31)			
Defined scope of efforts	1	1	3
Science-based approach to prevention	1	2	1
Sustainability planning to support prevention efforts	1	1	0
Created implementation plans for prevention strategies	0	0	1
Coalition connection with the program	0	0	1
Implementation support for programs	0	0	1

Additional details are provided in Table 3 Supplemental Information

**Table 4:** Coalition capacity characteristics never selected at any CoCU implementation year

Category (selected/total) Characteristic	Definition [*Describes*…]	
1. Mobilization (1/9)		
Support for prevention	How much the community supports preventing or addressing substance use, including whether a problem exists, how widely a prevention vision is shared, and whether leaders are willing to commit resources to reducing substance use	
2. Structure (14/21)		
Number of members	Number of members on the coalition roster	
Exit rate	Percentage of members who became inactive or stopped attending over the past 12 months [(members that left over last 12 months) ÷ (current members + members that left over last 12 months)]	
New member rate	Percentage of members who joined the coalition in the past 12 months (new members ÷ total members)	
Coalition staff stability	Difficulties hiring or retaining coalition staff, such as community mobilizers or program directors	
Communication	Quality of communication among members and leaders, including frequency, clarity, accuracy, and timeliness	
Shared understanding	How well members take the time to come to a common understanding on topics such as diverse perspectives, needs, values, and interests of all members	
Inclusive decision-making	How involved coalition members are in making decisions, including all members having a voice in the decision-making process and support of major decisions	
Number of meetings	Number of full coalition meetings in the past 12 months	
Challenges with transportation	How much of a problem it was to get to and from coalition meetings in the last year	
Sectoral diversity	Number of different agencies and institutions involved in the coalition compared to the number of members	
Distribution of information (centralization)	How much information, expertise, and influence were distributed across coalition members rather than controlled by a small number of members	
Cross-agency communication	How much communication occurs between coalition members from different community agencies or sectors	
Other organizations to engage in coalition’s efforts	Specific names of organizations members would like to be more actively supportive of or engaged with the coalition	
Cooperation with other groups	How much of a problem it was in the last year to obtain needed cooperation from other groups and agencies	
3. Leadership (0/2)		
4. Teamwork (2/5)		
Time spent on coalition activities	Average hours each coalition member gave to the coalition during a typical month over the past year, including time spent in full coalition, subcommittee, or workgroup meetings, and time spent outside of these meetings. ​	
Perception of the time spent on coalition activities	Members’ perception of the amount of time spent on coalition activities	
5. Planning and implementation (26/31)		
Resource availability	Coalition’s access to resources and supplies, including money and meeting space	
Future continuation	Members’ perception of coalition continuation over the next 3 years	
Financing plan	Degree to which the coalition developed a financing plan that takes into consideration program costs and other needs, such as staff time and office space	
Coalition funding for operations and activities	Funding sources, funding start and end dates, and the total amount of funds to support coalition operations and activities from federal, state, and local governments, foundations, businesses, and other sources	
Availability of PAYS data	School districts or schools within the coalition’s service area that participated in the latest administration of the Pennsylvania Youth Survey	
Identified risk and protective factors	Degree to which the coalition reviewed data and identified risk/protective factors and outcomes to prioritize for prevention efforts	
Documented plans to prevent substance use	Degree to which the coalition documented its planning process, decisions, and implementation plan for prioritized efforts to prevent substance use	
Annual review of prevention efforts	Degree to which coalition efforts to prevent substance use are evaluated at least annually, including discussing results and deciding next steps	
Number of effective and promising programs	Number of prevention programs supported by the coalition with sufficient level of evidence to be classified as either effective or promising	
Program staff training	Amount of training program staff received over the past 12 months	
Program staff motivation and skills	Level of staff engagement with the program over the past 12 months	
Coalition financial support for programming	How much financial support the coalition provides the program	
Program implementation barriers	Challenges that negatively impact successful delivery of the program	
Rigor of program fidelity monitoring	Quality and strength of program fidelity monitoring activities	
Breadth of program implementation monitoring	Number of program implementation factors monitored	
Adherence (amount of program content delivered)	How much of the prescribed program content (for youth and/or parents) from each program lesson was delivered as intended	
Program hours per participant (i.e., dosage)	Number of hours program participants spend connected to the program	
Program adaptation	Changes in the original program design to better fit participant needs, reflect cultural sensitivity, or other reasons	
Program reach	Extent to which a program attracts its intended audience	
Program outcome evaluation rigor	Quality and strength of program outcome evaluation activities	
Program evaluation report use	How program evaluation results are shared	
Fiscal planning for program implementation	Planning that has occurred to secure financial resources needed to support future implementation of the program	
Program promotion	Efforts to share information about the program with key stakeholders that could help sustain the program and strategies to identify key stakeholders who might support the program	
Organizational integration of program	Progress toward integrating the program into the everyday operations and budget of existing organizations; and plans to make the program a line-item in the budget of an organization, school, or community	
Program funding	Monetary funding for the program provided by different sources over certain time periods	

Additional details are provided in Table 4 Supplemental Information

Table 5 displays the results for the best longitudinal model, which includes the model fit statistics for the baseline, random intercept-only (i.e., empty) model, and the next best-fitting models. The random intercept time linear model reported lower AIC and BIC values with significant fixed effects, offering a better balance of fit and parsimony to describe the effect of time on scores of coalition capacity characteristics for each implementation year.

**Table 5:** Standardized growth estimates over time on characteristics selected for action planning

Implementation year	*β*	CI	*p*
Year 1 model (*n* = 32)^a^	0.13	0.004–0.26	.04
Year 2 model (*n* = 26)^b^	0.20	0.02–0.37	.03
Year 3 model (*n* = 25)^c^	0.61	0.16–1.07	.01

Data collection occurred four times: at the start of each of the three implementation years and a follow-up at the end of year 3

^a^Year 1 model assessed four timepoints—years 1 (baseline), 2, and 3, and follow-up after year 3

^b^Year 2 model assessed three timepoints—years 2 (baseline) and 3 and follow-up after year 3

^c^Year 3 model assessed two timepoints—year 3 (baseline) and follow-up after year 3

Consistent with our hypothesis, standardized growth estimates on self-identified characteristics of coalition capacity prioritized for action planning each year significantly improved over time as shown in Table 5. Initially prioritized capacities significantly improved in the first year (*β* = 0.13, 95% confidence interval (CI) [0.004, 0.26]; *p* =.04), as did priorities in the second year (*β* = 0.20, CI [0.02, 0.37]; *p* =.03) and in the third year (*β* = 0.61, CI [0.16, 1.07]; *p* =.01).

## Discussion

This study examined 3 years of Coalition Check-Up data to assess whether capacities coalitions selected for annual action planning improved through a low intensity data-driven TA model. As the first test of the CoCU model, the study focused on how the process functions: coalitions selected a single, general capacity characteristic to address over a defined period, while continuing their usual prevention activities. Results were consistent with our hypothesis: the capacities coalitions prioritized for action planning showed measurable improvement over time. We also found evidence suggesting that coalitions became more effective in their action-planning processes across implementation years. These findings suggest that coalition-identified capacities strengthened over the course of the CoCU intervention during interactions with the TA provider. By selecting their own goals and areas for growth, coalitions engaged in a structured process that aligns with broader evidence showing that TA can enhance coalition functioning (Chilenski et al., 2021). Overall, the results imply that a low intensity TA model can support ongoing capacity development, with important implications for future research and refinement of scalable TA approaches for community coalitions. Although CoCU requires active engagement from both TA providers and coalitions, it reflects a lower-dosage model (16 TA encounters per year) relative to more intensive TA approaches (e.g., ~ 0.25 FTE) (Chilenski et al., 2021). Alongside other previously published analyses of the CoCU (Chilenski et al., 2026; Gaddy et al., 2024; Gaddy et al., 2025; Im et al., 2025; Wells et al., 2026), future work needs to link these observed proximal CoCU TA outcomes to later downstream effects on implementation of evidence-based programs, their implementation quality, and youth substance use.

Examining the trajectory of change across years, we found that the strength of the association of time was stronger in the years with shorter follow-up timeframes. This could be an artifact of longitudinal data, where more measurements resulted in a smoothing of scores over time, as might be more evident for year 1. That is, priorities selected at year 1 were assessed three additional times, while those selected in year 3 only had two measurements—at the beginning and at the end of year 3. However, a linear model, not a quadratic model, best fit the year 1 selected priorities. A linear as opposed to a quadratic model suggests the lack of systematic change in slope across the four time points. That said, we would expect that, at some point, the rate of change would slow and level off. The improved slopes over time could be an indication that coalitions got better at building and implementing their action plans to improve their selected capacities. Alternatively, TA providers and coalitions may have improved over time in aligning action planning with capacity-building efforts. Coalition-specific factors, such as leadership turnover, restructuring, or developmental stage, or even historical factors, such as the COVID-19 pandemic, may also have impacted the rate of change over time (Chilenski et al., 2021; Christian et al., 2022; Feinberg et al., 2008c; Greenberg et al., 2007; Katz & Wandersman, 2016).

Our findings also revealed consistent patterns in the types of capacities coalitions selected. Coalitions tended to prioritize mobilization-related capacities, which is consistent with the thesis that mobilization is the backbone to coalition effectiveness (Butterfoss & Kegler, 2002; Foster-Fishman et al., 2001). This aligns with prior research showing that coalitions mobilized around shared priorities, member and community engagement, and supported by TA are more likely to sustain momentum, adapt to change, and achieve long-term impact (Chilenski et al., 2019; Florin et al., 1993; Keene Woods et al., 2014). Prior research indicates that mobilized coalitions also tend to share resources (Gaddy et al., 2024; Scott et al., 2022)—especially critical for newer, smaller, or resource-constrained coalitions—and community-driven mobilization enhances collaboration and program delivery (Nagorcka-Smith et al., 2022).

We also observed consistent patterns in the types of capacities coalitions tended not to select. Notably, few program-related capacities were selected, even though yearly assessments included program planning and implementation capacities. Given the importance of programming for positive youth development, particularly evidence-based programs (Mihalic & Elliott, 2015), this low prioritization may warrant future attention. The infrequency with which leadership was selected similarly merits consideration, given prior evidence of its central role in coalition functioning (Birgel et al., 2025; Brown et al., 2015; Florin et al., 1993; Greenberg et al., 2007).

Interestingly, priority selection patterns shifted over time. For instance, “developed mission and communication plan” was selected in years 1 and 2, but not in year 3. Given that a number of coalitions had already been in operation for at least 1 year at the start of the study, it is possible that subsequent shifts in selections may have been influenced by major events, such as leadership changes, member turnover, coalition mergers, or the impact of COVID-19 on coalition operations and rebuilding efforts (Greenberg et al., 2007; Imm et al., 2020). It might also be that coalitions shifted from a need for mobilization and structure, which is more foundational, to more readiness for planning and implementation, which is more directly related to youth outcomes.

### Implications

Taken together, these findings indicate that the CoCU TA model—a low intensity, data-driven, collaborative approach using motivational interviewing techniques—has the potential to strengthen coalition capacity development over time. Models such as CoCU may broaden access to TA, making it more feasible for coalitions, particularly those with limited resources. Coalitions, TA providers, and funders should weigh the benefits of sustained TA against the resource costs, investing in evidence-based approaches to TA that promote sustainable capacity development. Though beyond the scope of the current analyses, it would also be worthwhile to investigate if and how the priority selection and action planning skills developed through the CoCU process generalized to other coalition decisions and activities. Generalizing these skills to other coalition decisions and activities would increase the relative gain compared to the costs of implementation.

Refinements to the CoCU model may be warranted. Only 26 of 68 coalition capacity and program implementation characteristics were selected across the 3 years. Streamlining the assessment by reducing the number of characteristics could lessen the burden of data collection while maintaining relevance. Another option to reduce data collection burden might be to remove program coordinator input but keep items related to program planning and implementation in the coalition leader interview and member survey. Given the importance of basing prevention on evidence, future efforts could integrate key community health assessment data into the feedback summary or even explore ways to gently guide coalitions to prioritize characteristics related to evidence-based practices.

### Limitations

Though this study was larger (*n* = 32 intervention) than many other studies of coalitions, such as PROSPER (*n* = 14 intervention) (Greenberg et al., 2007), Communities That Care (*n* = 12 intervention) (Arthur et al., 2010), and Getting to Outcomes (*n* = 2 intervention) (Chinman et al., 2008), this study involved a limited sample of coalitions, and findings should be cautiously interpreted. The final sample may also have become more representative of stronger coalitions than the initial sample. By year 3, three of the 32 coalitions in the initial sample dropped from the study, some of which may have become inoperative. Four of the remaining 29 coalitions at that point did not identify priorities due to leadership changes or loss of funding. Hence, by year 3, weaker coalitions may have dropped out or disbanded, leaving only stronger coalitions that were more likely to improve their selected capacities. Furthermore, given that the comparison group did not select priorities for action planning, the absence of a comparison group for these analyses limits causal inference. Future research could explore ways to work with the comparison group to create a comparable and testable control sample for these analyses. Doing so may also enable future efforts to assess what impact, if any, CoCU had on coalitions’ decisions—intervention and comparison—in subsequent years, and whether CoCU influenced EBP implementation, particularly among coalitions that did not previously implement EBPs. Additionally, we are conducting a cost and dosage comparison relative to more intensive TA models, which we do not yet have (Chilenski et al., 2021). Lastly, bias may have occurred if coalitions chose easier goals or were influenced by their awareness of being assessed (e.g., Hawthorne effect (Perera, 2024)).

## Conclusions

Coalitions receiving hands-on, collaborative, and structured TA support through the CoCU model exhibited measurable improvements in self-identified capacities over time. These findings, rooted in the ISF (Wandersman et al., 2008), support the use of a low intensity TA model like the Coalition Check-Up to guide coalitions toward balanced and strategic general capacity development. This approach is theorized to enhance coalitions’ innovation-specific capacities, including their ability to deliver impactful, evidence-based prevention initiatives (Wells et al., 2026). Effects were short-term by design. The results demonstrate CoCU’s utility as a scalable, low intensity support-system intervention that strengthens conditions necessary for effective prevention efforts (Wandersman et al., 2008). Our findings underscore the benefits of continued investment in adaptable TA models to reinforce coalitions’ strength, resilience, and capacity for long-term success, even among sustained coalitions.

## Supplementary Information

Below is the link to the electronic supplementary material.

ESM 1 (DOCX 68.0 KB)

## Data Availability

The data that supports the findings of this study are available from the corresponding author upon reasonable request.
