# Supplementary material for: Improvements in Capacity Among Community Coalitions Receiving Technical Assistance Through the Coalition Check-Up
Source: Prev Sci. 2026 Jun 24;27(6):958–70. doi: 10.1007/s11121-026-01941-z (PMC13433725; doi:10.1007/s11121-026-01941-z)
Supplement: Supplementary file 1 [file 11121_2026_1941_MOESM1_ESM.docx]

**Supplemental Information**

**Characteristics of Coalition Capacity**

**Coalition Check-Up (CoCU)**

The following tables provide details on 68 characteristics of coalition capacity and program implementation from the CoCU study, which we grouped into five general categories (Nargiso et al., 2013):

1) mobilization - capacity to harness the collective power of a wide range of community partners to work together toward common goals

2) structure - capacity to establish a formalized structure to maximize operations

3) leadership - capacity for leadership that promotes vital action for coalition operations

4) teamwork - capacity to foster synergistic collaboration

5) planning and implementation - capacity to establish plans to support optimal implementation and sustainability of prevention efforts.

Table 3 Supplemental Information provides additional information on the characteristics selected for action planning, as described in Table 3 of the manuscript. Table 4 Supplemental Information includes additional details on the characteristics coalitions never selected during the study, as shown in Table 4 of the manuscript.

Nargiso, J. E., Friend, K. B., Egan, C., Florin, P., Stevenson, J., Amodei, B., & Barovier, L. (2013). Coalitional capacities and environmental strategies to prevent underage drinking. *American Journal of Community Psychology*, *51*(1-2), 222–231. <https://doi.org/10.1007/s10464-012-9536-4>

**Table 3 Supplemental Information**

*Details on characteristics of coalition capacity selected for action planning at any CoCU implementation year (as described in Table 3 of the manuscript)*

| **Category (selected / total)**  **Characteristic** | **Definition [*Describes*…]** | **# of Items** | **M, L, PC ^a^** | **α ^b^** | **Sample Item** | **Response Options** |
| --- | --- | --- | --- | --- | --- | --- |
| 1. Mobilization (8 / 9) | | | | | | |
| Member recruitment | Difficulty getting people with the needed skills, talents, and connections to join the coalition. | 1 | M | -- | In the past 12 months, how much of a problem was getting the "right" people to join the coalition; that is, people with the skills, talents, or political connections the coalition needed? | 1 = Huge problem  2 = Big problem  3 = Moderate problem  4 = Slight problem  5 = Not a problem |
| Community knowledge of the coalition's work | Degree to which people who live and work in the community are knowledgeable about the coalition’s work. | 1 | L | -- | To what degree are the people that live and work in the area served by your coalition boundaries knowledgeable about your coalition's work? | 0 = Not at all  1 = A little  2 = Somewhat  3 = Mostly  4 = Completely |
| Inclusion of community residents | How well the coalition considers the perspective of those affected by the coalition’s decisions, including seeking out and facilitating their participation and viewpoints. | 2 | M | *r=*.80 | How much do you agree or disagree that this coalition seeks out and facilitates the participation of individuals affected by the coalition's decisions? | 1 = Strongly disagree  2 = Disagree  3 = Neutral  4 = Agree  5 = Strongly agree |
| School support for the coalition | Involvement of school personnel with the coalition, including their commitment and whether a champion for the coalition exists within the school system. | 4 | M | .88 | Please rate how well influential school leaders understand your coalition and why it is important. | 1 = Not at all  2 = A little  3 = Some  4 = A good amount  5 = A great deal |
| Clarity of internal coalition roles | How well coalition responsibilities for members are defined and how well expectations are communicated. | 3 | M | .96 | How much do you agree or disagree that your role within your coalition is clearly defined? | 1 = Strongly disagree  2 = Disagree  3 = Neutral  4 = Agree  5 = Strongly agree |
| Community support for the coalition | Involvement of community leaders with the coalition and how well they understand the coalition. | 3 | M | .87 | Please rate how well influential community leaders understand your coalition and why it is important. | 1 = Not at all  2 = A little  3 = Some  4 = A good amount  5 = A great deal |
| Number of member roles | Roles each coalition member played in the past 12 months. | 1 | M | --- | In the past 12 months, did you 1) talk at meetings, 2) serve as a member of a committee, 3) chair/lead a committee or group, 4) undertake some other one-time or 5) long-term responsibility? | *Number of positive responses* |
| Member involvement | Difficulty getting members to commit and work hard on coalition goals. | 1 | M | --- | In the past 12 months, how much of a problem was it to get coalition members to make a strong commitment or work hard on coalition goals. | 1 = Huge problem  2 = Big problem  3 = Moderate problem  4 = Slight problem  5 = Not a problem |
| 2. Structure (7 / 21) | | | | | | |
| Developed mission & communications plan | Degree to which the coalition has finalized a vision and mission statement, and tools and strategies to communicate with outside groups. | 1 | L | --- | To what degree has your coalition finalized a vision or mission statement and developed tools and strategies to communicate with outside groups? | 0 = Not at all  1 = A little  2 = Somewhat  3 = Mostly  4 = Completely |
| Members oriented to the coalition’s implementation model | Degree to which the coalition oriented its current members to the implementation model. | 1 | L | --- | To what degree has your coalition oriented its current members to its selected implementation model? | 0 = Not at all  1 = A little  2 = Somewhat  3 = Mostly  4 = Completely |
| Number of committee, subcommittee, and/or workgroup meetings | Number of committee, subcommittee, or workgroup meetings in the past 12 months. | 1 | L | --- | How many committees, subcommittees, or workgroups held meetings in the past 12 months? | *Number* |
| Formalized structure for consistent implementation of policies and procedures | Degree to which the coalition has formalized its structure, considering how consistently its policies and procedures were implemented. | 1 | L | --- | To what degree has your coalition formalized its structure, including how consistent your policies and procedures are implemented. | 0 = Not at all  1 = A little  2 = Somewhat  3 = Mostly  4 = Completely |
| Attendance | Average number of members that attended each coalition meeting. | 1 | L | --- | On average, how many people attended each coalition meeting in the last 12 months? | 1 = 1-4  2 = 5-9  3 = 10-14  4 = 15-20  5 = 21-30  6 = 31+ |
| Member collaboration with different organizations ^c^ | Member collaboration with other community agencies [*22 listed*] to share information, monetary resources, personnel, and other types of cooperation. | 22 | M | --- | Do you or your workplace work with schools to [*response options*] on youth substance abuse prevention? Check all responses that apply regardless of whether it is coalition related. | 1 = Share information outside coalition meetings  2 = Share monetary resources  3 = Share personnel  4 = Other cooperation (not information, monetary, or personnel) |
| External partner role clarity | Degree to which the coalition has clarified the roles of coalition members, provider/contracted agencies, and other volunteers in implementing and evaluating programs and activities. | 1 | L | --- | To what degree has your coalition clarified the roles of coalition members, provider/contracted agencies, or other volunteers in implementing and evaluating its selected programs or activities? | 0 = Not at all  1 = A little  2 = Somewhat  3 = Mostly  4 = Completely |
| 3. Leadership (2 / 2) | | | | | | |
| Number of formal leadership positions | Number of formal leadership positions on the coalition. | 1 | L | --- | How many formal leadership positions are there on your coalition (e.g., chair, officers, committee chairs)? | *Number* |
| Leadership transitions | Number of leadership positions that changed hands from one member to another in the last 12 months. | 1 | L | --- | How many leadership positions changed hands from one member to another in the last 12 months? | *Number* |
| 4. Teamwork (3 / 5) | | | | | | |
| Cohesion | Coalition connection among members, including unity, cohesion, trust, and closeness to each other. | 3 | M | .90 | How much do you agree or disagree that within your coalition, there is a feeling of unity and cohesion? | 1 = Strongly disagree  2 = Disagree  3 = Neutral  4 = Agree  5 = Strongly agree |
| Meeting focus | Smoothness coalition moves through and accomplishes meeting agenda items, including having clear meeting goals and well-managed discussions focused on high-priority issues. | 3 | M | .79 | How much do you agree or disagree that the goals of the meetings are not always clear? | 1 = Strongly agree  2 = Agree  3 = Neutral  4 = Disagree  5 = Strongly disagree |
| Efficient meetings | Coalition work ethic and output, including member dedication and how efficient and work-oriented members are at meetings. | 3 | M | .89 | How much do you agree or disagree that this coalition is highly efficient and work-oriented? | 1 = Strongly disagree  2 = Disagree  3 = Neutral  4 = Agree  5 = Strongly agree |
| 5. Planning and implementation (6 / 31) | | | | | | |
| Defined scope of efforts | How well the coalition has defined the area selected for intervention and how to interface with other community initiatives. | 1 | L | --- | To what degree has your coalition defined the scope of the prevention effort, considering how well you have defined the area selected for intervention and discussed how you will interface with existing community initiatives? | 0 = Not at all  1 = A little  2 = Somewhat  3 = Mostly  4 = Completely |
| Science-based approach to prevention | Use of information from previous research and ongoing evaluation to make decisions about youth and family programs. | 6 | M | .78 | In the past 1-2 years, how often did your coalition evaluate if programs affect risk and protective factors levels among the individuals participating in these programs? | 0 = Never  1 = Almost never  2 = A little  3 = Somewhat  4 = Often  5 = Usually  6 = Always |
| Created implementation plans for prevention strategies | Specification of personal, financial, and technical resources needed to implement each drug prevention strategy. | 1 | L | --- | To what degree has your coalition specified the human, financial, and technical resources needed to implement each selected drug prevention strategy with fidelity? | 0 = Not at all  1 = A little  2 = Somewhat  3 = Mostly  4 = Completely |
| Sustainability planning to support prevention efforts | How detailed the plans are to keep coalition activities and programs going, including the exploration of funding strategies and development and implementation of a realistic, concrete plan on how to continue offering programs and engaging volunteers. | 4 | M | .87 | To what degree has your coalition developed a realistic, concrete plan on how to continue offering programs? | 1 = Not at all  2 = A little  3 = Some  4 = A lot |
| Coalition connection with the program | How connected program facilitators or coordinators are to the coalition. | 3 | PC | .67 | How often does a representative of the program attend your coalition meetings? | 0 = None  1 = A little  2 = Somewhat  3 = Quite a bit  4 = A great deal |
| Implementation support for programs | Logistical support the coalition provides the program. | 8 | PC | .88 | In the past 12 months, to what extent has your coalition encouraged interagency collaboration? | 0 = None  1 = A little  2 = Somewhat  3 = Quite a bit  4 = A great deal |

^a^ L = Coalition leader interview; M = Member survey; PC = Program coordinator interview/survey

^b^ Cronbach’s alpha unless otherwise noted.

^c^ A list of 22 community sectors was provided, such as schools, health care organizations, law enforcement, and faith-based organizations. The complete list is shown in the notes below Table 4 Supplemental Information.

**Table 4 Supplemental Information**

*Details on characteristics of coalition capacity never selected during the CoCU study (as shown in Table 4 of the manuscript)*

| **Category (not selected / total)**  **Characteristic** | **Definition [*Describes*…]** | **# of Items** | **M, L, PC ^a^** | **α ^b^** | **Sample Item** | **Response Options** |
| --- | --- | --- | --- | --- | --- | --- |
| 1. Mobilization (1 / 9) | | | | | | |
| Support for prevention | Community support for preventing or addressing substance use, including whether a problem exists, how widely a prevention vision is shared, and whether leaders are willing to commit resources to reducing substance use. | 3 | M | .81 | In thinking about your coalition and the area it serves, how much do you agree or disagree that the community recognizes there are substance abuse problems that need to be addressed? | 1 = Strongly disagree  2 = Disagree  3 = Neutral  4 = Agree  5 = Strongly agree |
| 2. Structure (14 / 21) | | | | | | |
| Number of members | Number of coalition members. | 1 | L | --- | How many members are there on this coalition currently? | *Number* |
| Exit rate | Percentage of members who became inactive or stopped attending over the past 12 months [(inactive members last 12 months) ÷ (current members + inactive members last 12 months)] | 2 | L | *r=*.28 | How many members dropped out or became inactive in the last 12 months? | *Number* |
| New member rate | Percentage of members who joined the coalition in the past 12 months  (new members ÷ total members) | 2 | L | *r=*.39 | How many members joined the coalition in the last 12 months? | *Number* |
| Coalition staff stability | Difficulties hiring or retaining coalition staff, such as community mobilizers or program directors. | 1 | M | --- | In the past 12 months, how much of a problem was hiring and retaining high-quality staff (e.g., community mobilizer or program director)? | 1 = Huge problem  2 = Big problem  3 = Moderate problem  4 = Slight problem  5 = Not a problem |
| Communication | Quality of communication among members and leaders, including frequency, clarity, accuracy, and timeliness. | 4 | M | .93 | How would you rate the frequency of communication between coalition leaders and coalitions members? | 1 = Needs a lot of work  2 = Needs a little work  3 = Good  4 = Outstanding |
| Shared understanding | How well members take the time to come to a common understanding on topics such as diverse perspectives, needs, values, and interests of all members. | 3 | M | .89 | How much do you agree or disagree that when there are differences of opinion, this coalition takes time to fully understand each perspective? | 1 = Strongly disagree  2 = Disagree  3 = Neutral  4 = Agree  5 = Strongly agree |
| Inclusive decision-making | How involved members are in making decisions, including all members having a voice in the decision-making process and support of major decisions. | 3 | M | .89 | How much do you agree or disagree that all members have a fair say in coalition decisions? | 1 = Strongly disagree  2 = Disagree  3 = Neutral  4 = Agree  5 = Strongly agree |
| Number of meetings | Number of full coalition meetings in the past 12 months. | 1 | L | --- | About how many coalition meetings occurred in the past 12 months? (i.e., meetings with all coalition members, not subcommittee or workgroup meetings that include a subset of individuals from this full meeting) | *Number* |
| Challenges with transportation | How much of a problem it was to get to and from coalition meetings in the last year. | 1 | M | --- | In the past 12 months, how much of a problem was it for members to get to and from meetings (i.e., transportation difficulties)? | 1 = Huge problem  2 = Big problem  3 = Moderate problem  4 = Slight problem  5 = Not a problem |
| Sectoral diversity ^c,d^ | Number of different agencies and institutions involved in the coalition compared to the number of members. | 22 | M | --- | Of the following community sectors, which one best describes who you represent on this coalition? | *See table notes* |
| Distribution of information (centralization) | How much information, expertise, and influence were distributed across members rather than controlled by a small number of members. | 5 | M | --- | Whom do you approach for information or advice on coalition-related issues? List full names of up to five other members of this coalition in rank order in terms of whom you approach first, second, and so on, up to five. | *Text* |
| Cross-agency communication | How much communication occurs between coalition members from different community agencies or sectors. |  | *Calculations were based on the sector the respondent and each person they named represented on the coalition.* | | | |
| Other organizations to engage in coalition’s efforts | Organizations coalition members would like to be more actively supportive of or engaged with the coalition. | 1 | M | --- | Which specific organizations (affiliated or not) would you like to be more actively supportive of or engaged with the coalition? | *Text* |
| Cooperation with other groups | How much of a problem it was in the last year to obtain needed cooperation from other groups and agencies. | 1 | M | --- | In the past 12 months, how much of a problem was it to obtain sufficient cooperation from other groups and agencies to achieve coalition goals? | 1 = Huge problem  2 = Big problem  3 = Moderate problem  4 = Slight problem  5 = Not a problem |
| 3. Leadership (0 / 2 | | | | | | |
| 4. Teamwork (2 / 5) | | | | | | |
| Time spent in coalition activities | Average hours each member gave to the coalition during a typical month over the past year, including time spent in full coalition, subcommittee, or workgroup meetings, and time spent outside of these meetings. | 2 | M | *r=*.49 | During the past year, on average, how many hours per month did you spend in coalition meetings, including full coalition, subcommittee, or workgroup meetings? | *Number* |
| Perception of the time spent on coalition activities | Members’ perception of the amount of time spent on coalition activities. | 1 | M | *---* | Overall, how would you describe the amount of time you spent on coalition activities over the past year? | 1 = Too much  2 = Just about right  3 = Too little |
| 5. Planning and implementation (26 / 31) | | | | | | |
| Resource availability | Coalition’s access to resources and supplies, including money and meeting space. | 4 | M | .88 | How much do you agree or disagree that scarcity of resources is a real problem for this coalition? | 1 = Strongly agree  2 = Agree  3 = Neutral  4 = Disagree  5 = Strongly disagree |
| Future continuation | Members’ perception of coalition continuation over the next 3 years. | 1 | M | --- | How likely do you think it is that your coalition will continue for the next 3 years? | 1 = Highly unlikely  2 = Somewhat unlikely  3 = Somewhat likely  4 = Highly likely |
| Financing plan | Degree to which the coalition developed a financing plan that takes into consideration program costs and other needs, such as staff time and office space. | 1 | L | --- | To what degree has your coalition developed a financing plan, including identified and projected financial needs to support sustainability of programs? | 1 = Not at all  2 = A little  3 = Some  4 = A lot |
| Coalition funding for operations & activities | Funding sources, funding start and end dates, and the total amount of funds to support coalition operations and activities from federal, state, and local governments, foundations, businesses, and other sources | 1 | L | --- | What was the total amount of the award the coalition received from [*source*]? | *Monetary amount* |
| Availability of PAYS data | School districts or schools within the coalition’s service area that participated in the latest administration of the Pennsylvania Youth Survey. | 1 | --- | --- | List of school districts or schools within the coalition’s service area that participated in the latest administration of the PAYS | |
| Identified risk & protective factors | Degree to which the coalition reviewed data and identified risk/protective factors and outcomes to prioritize for programming. | 1 | L | --- | To what degree has the coalition identified which risk/protective factors and outcomes to prioritize for programming, based on reviewing youth and community data? | 0 = Not at all  1 = A little  2 = Somewhat  3 = Mostly  4 = Completely |
| Documented plans to prevent substance use | Degree to which the coalition documented its planning process, decisions, and implementation plan for prioritized efforts to prevent substance use. | 1 | L | --- | To what degree has your coalition documented its planning process, decisions, and implementation plan? | 0 = Not at all  1 = A little  2 = Somewhat  3 = Mostly  4 = Completely |
| Annual review of prevention efforts | Degree to which coalition efforts to prevent substance use are evaluated at least annually, including discussing results and deciding next steps | 1 | L | --- | To what degree are coalition efforts to prevent substance use evaluated at least annually, including discussing findings and making decisions about next steps after reviewing the data? | 0 = Not at all  1 = A little  2 = Somewhat  3 = Mostly  4 = Completely |
| Number of effective and promising programs | Number of prevention programs supported by the coalition with sufficient level of evidence to be classified as either effective or promising. | 1 | --- | --- | *Number* | |
| Program staff training | Amount of training program staff received over the past 12 months. | 5 | PC | .35 | Of the staff who have implemented [*program*] in the past 12 months, how many received formal training to implement the program? | 1 = None  2 = A few  3 = Less than half  4 = About half  5 = More than half  6 = Almost all  7 = All |
| Program staff motivation & skills | Level of staff engagement with the program over the past 12 months. | 3 | PC | .84 | Please indicate how much you agree or disagree that the individuals who implemented [*program*] over the past 12 months had the required skills and knowledge of the program model and key elements. | 1 = Strongly disagree  2 = Disagree  3 = Neutral  4 = Agree  5 = Strongly agree |
| Coalition financial support for programming | How much financial support the coalition provides the program. | 3 | L | .71 | In the past 12 months, to what extent has your coalition assisted in seeking funding for [*program*], including identifying funding sources or helping with a grant application? | 1 = Not at all  2 = A little  3 = Some  4 = A good amount  5 = A great deal |
| Program implementation barriers | Challenges that negatively impact successful delivery of the program. | 8 | PC | .67 | How much did the difficulty in recruiting participants hinder [*program*] implementation during the past 12 months? | 1 = Huge problem  2 = Big problem  3 = Moderate problem  4 = Slight problem  5 = Not a problem |
| Rigor of program fidelity monitoring | Quality and strength of fidelity monitoring activities. | 3 | PC | .66 | In the last 12 months, did you use EPIS or program developer fidelity materials? | 0 = No  1 = Yes |
| Breadth of program implementation monitoring | Number of program implementation factors monitored. | 6 | PC | .84 | Did you monitor [*program*] implementation by collecting data on the number of sessions held in the past 12 months? | 0 = No  1 = Yes |
| Adherence (amount of content delivered) | How much of the prescribed program content (for parents and/or youth) from each program lesson was delivered as intended. | 2 | PC | *r=*.76 | In the last 12 months, on average, when [*program*] was delivered, how much of the outlined content was actually delivered? | 1 = None  2 = A little  3 = Less than half  4 = About half  5 = More than half  6 = Almost all  7 = All |
| Program hours per participant (i.e., dosage) | Number of hours program participants spend connected to the program. | 1 | PC | --- | How many lessons does a parent or youth typically participate in and how long is a typical lesson for [*program*]? | *Number (lessons x minutes)* |
| Program adaptation | Changes in the original program design to better fit participant needs, reflect cultural sensitivity, or other reasons. | 5 | PC | .59 | Please indicate to what extent changes in procedures, such as the location, time, or recruitment process, were made over the last 12 months. | 1 = No change  2 = Minor change  3 = Moderate change  4 = Major change |
| Program reach | Extent to which a program attracts its intended audience. | 1 | PC | --- | How many youth did [*program*] reach at this location in the past 12 months? | *Number* |
| Program outcome evaluation rigor | Quality and strength of outcome evaluation activities. | 5 | PC | .41 | Is data being collected about participant outcomes before they start [*program*]? | 0 = No  1 = Yes |
| Program evaluation report use | How evaluation results are shared. | 4 | PC | .66 | In the past 12 months, have you or someone you work with developed a specific report describing [*program*] impact/outcomes? | 0 = No  1 = Yes |
| Fiscal planning for program implementation | Planning that has occurred to secure financial resources needed to support future implementation of the program. | 6 | PC | .71 | Please indicate the degree to which you planned to apply for additional grants over the past 12 months to actively support sustainability of [*program*]. | 1 = No plan  2 = Tentative plan  3 = Clear plan, no implementation  4 = Plan with initial implementation  5 = Plan with extensive implementation |
| Program promotion | Efforts taken to share information about the program with key stakeholders that could help sustain the program and strategies to identify key stakeholders who might support the program. | 4 | PC | .85 | Please indicate the degree to which you planned to present outcome data to potential stakeholders over the past 12 months to actively support sustainability of [*program*]. | 1 = No plan  2 = Tentative plan  3 = Clear plan, no implementation  4 = Plan with initial implementation  5 = Plan with extensive implementation |
| Organizational integration of program | Progress toward integrating the program into the everyday operations and budget of existing organizations; and plans to make the program a line- item in the budget of your organization, school, or community. | 2 | PC | *r=*.42 | Please indicate the degree to which you planned to identify strategies to integrate the program into existing organizations, agencies, schools, and/or communities over the past 12 months to actively support sustainability of [*program*]. | 1 = No plan  2 = Tentative plan  3 = Clear plan, no implementation  4 = Plan with initial implementation  5 = Plan with extensive implementation |
| Program funding | Monetary funding for the program provided by different sources over certain time periods. | 1 | PC | --- | List a source for [*program*] funding in the past 12 months, including start and end dates, and total funding. | *Monetary amount* |

^a^ L = Coalition leader interview; M = Member survey; PC = Program coordinator interview/survey

^b^ Cronbach’s alpha unless otherwise noted.

^c^ List of community sectors:

1 = Elementary, Middle, or High school(s), school districts

2 = Other education (e.g., universities, technical colleges)

3 = Media (e.g., newspapers, TV stations)

4 = Business (e.g., chamber of commerce, transportation companies)

5 = Law enforcement

6 = Judicial System (e.g., Juvenile Justice, Family Court, etc.)

7 = Firefighters or paramedics

8 = Religious / Faith Organization

9 = Civic or volunteer groups (e.g., Lions Club, Rotary Club, philanthropy)

10 = Local government (i.e., city/county) Parks and Recreation

11 = Youth serving organizations (e.g., YMCA, Boys & Girls Clubs, Communities in Schools, other youth activities)

12 = Child Welfare

13 = Public Health Department (Local or State)

14 = Substance use-related organizations (e.g., prevention or treatment, public or private)

15 = Mental / behavioral health services (e.g., prevention or treatment, public or private)

16 = Healthcare providers, hospitals, or other health organizations

17 = Cooperative Extension

18 = Other federal or state government agencies

19 = Other local government

20 = Child / Family Advocacy

21 = Unaffiliated parent or community resident

22 = Youth (any individual under 18)

^d^ Scores calculated using an entropy index (Gaddy et al., 2024; Ramaciotti Morales et al., 2021).

Gaddy, Y., Jones, E. C., Wells, R., Chilenski, S. M., & Brown, L. D. (2024). Effects of resource sharing networks on community anti-drug coalitions’ outcomes: A social network analysis. *Prevention Science*. <https://doi.org/10.1007/s11121-024-01719-1>

Ramaciotti Morales, P., Lamarche-Perrin, R., Fournier-S'niehotta, R., Poulain, R., Tabourier, L., & Tarissan, F. (2021). Measuring diversity in heterogeneous information networks. *Theoretical Computer Science*, *859*, 80–115. <https://doi.org/10.1016/j.tcs.2021.01.013>
